# Target of rapamycin, *PvTOR*, is a key regulator of arbuscule development during mycorrhizal symbiosis in *Phaseolus*

**DOI:** 10.1038/s41598-021-90288-2

**Published:** 2021-05-31

**Authors:** Manoj-Kumar Arthikala, Kalpana Nanjareddy, Lourdes Blanco, Xóchitl Alvarado-Affantranger, Miguel Lara

**Affiliations:** 1 Ciencias Agrogenómicas, Escuela Nacional de Estudios Superiores Unidad León-Universidad Nacional Autónoma de México (UNAM), C.P. 37689 León, Guanajuato México; 2 Departamento de Biología Molecular de Plantas, Instituto de Biotecnología, UNAM, C.P. 62210 Cuernavaca, México; 3 Laboratorio Nacional de Microscopía Avanzada, Instituto de Biotecnología, UNAM, C.P. 62210 Cuernavaca, México

**Keywords:** Plant sciences, Plant development, Plant molecular biology, Plant signalling, Plant symbiosis

## Abstract

Target of rapamycin (TOR) is a conserved central growth regulator in eukaryotes that has a key role in maintaining cellular nutrient and energy status. Arbuscular mycorrhizal (AM) fungi are mutualistic symbionts that assist the plant in increasing nutrient absorption from the rhizosphere. However, the role of legume TOR in AM fungal symbiosis development has not been investigated. In this study, we examined the function of legume TOR in the development and formation of AM fungal symbiosis. RNA-interference-mediated knockdown of *TOR* transcripts in common bean (*Phaseolus vulgaris*) hairy roots notably suppressed AM fungus-induced lateral root formation by altering the expression of root meristem regulatory genes, i.e., *UPB1*, RGFs, and sulfur assimilation and S-phase genes. Mycorrhized *PvTOR*-knockdown roots had significantly more extraradical hyphae and hyphopodia than the control (empty vector) roots. Strong promoter activity of *PvTOR* was observed at the site of hyphal penetration and colonization. Colonization along the root length was affected in mycorrhized *PvTOR*-knockdown roots and the arbuscules were stunted. Furthermore, the expression of genes induced by AM symbiosis such as *SWEET1*, *VPY*, *VAMP713*, and *STR* was repressed under mycorrhized conditions in *PvTOR*-knockdown roots. Based on these observations, we conclude that *PvTOR* is a key player in regulating arbuscule development during AM symbiosis in *P. vulgaris*. These results provide insight into legume TOR as a potential regulatory factor influencing the symbiotic associations of *P. vulgaris* and other legumes.

## Introduction

Arbuscular mycorrhizal (AM) fungi, obligate biotrophs belonging to the phylum Mucoromycota and subphylum Glomeromycotina^1^, are the most ancient and perhaps the most important group of symbionts on earth. Fossil records and molecular clock dating indicate that AM fungal associations emerged 460 million years ago^2^, supporting the idea that the ability of plants to form these associations is one of the most remarkable and enduring adaptations to life on land. The vast majority of vascular plants can form symbioses with AM fungi^3^, which help the host plants take up and translocate macronutrients (phosphorus [P] and nitrogen [N]), micronutrients, and water from the soil^4^. In return, the host plant fulfills the organic carbon^5^ and lipid^6,7^ requirements of the AM fungi. AM fungi also protect against pathogenic fungi and diverse abiotic stresses^8^, making them of great interest for sustainable agriculture^9^.

The mutualistic relationship between plants and AM fungi begins in the rhizosphere with a molecular dialogue between the partners. In response to strigolactones in the host root exudate^10^, the germinating AM fungal spores synthesize and secrete Myc factors^11^. Chitooligosaccharides (COs) and lipo-chitooligosaccharides (LCOs), that promote symbiosis signaling by inducing oscillations in nuclear-associated calcium and inducing the expression of AM fungi-responsive plant genes that trigger phenotypic changes in the host roots^11,12^. The development of fungal hyphopodia establishes cell-to-cell contact between the host root and the AM fungus, thus initiate the common symbiotic signaling pathway (CSSP). Hyphal invasion and colonization of the root involves formation of an infection peg from the hyphopodium, which mediates hyphal growth into root epidermal cells, and then a prepenetration apparatus, which dictates the intracellular routes by which the fungus traverses the epidermis^12^. The fungal hyphae grow and ramify within the intra- and intercellular spaces of the root cortex and subsequently invade the inner cortical cells. Within these cells, the fungal hyphae develop finely branched structures called arbuscules. Each arbuscule remains separated from the plant cell cytoplasm by a plant-derived extension of the plasma membrane called the periarbuscular membrane. This membrane surrounds the hyphal branches and constitutes an interface that is specialized for the exchange of nutrients between the partners^13^. Given the importance of AM symbiosis, the signaling networks that underlie mycorrhizal interactions have been well studied. So far, several genes and transcription factors have been reported to be essential for AM symbiotic associations^14^ such as *RAM1*, *RAD1*, *MIG1*, *ERF1*, *RAM2*, *STR1/2*, *PT4* and *PT11* to name a few.

Target of rapamycin (TOR), a phosphatidylinositol kinase-related protein kinase, is functionally and structurally conserved from yeast to animals and plants^15^. TOR plays a key role in maintaining cellular nutrient and energy status, controlling the activity, localization, and stability of its target proteins by phosphorylation of serine and/or threonine residues^16^. In mammals and plants, TOR is encoded by a single gene, while fungi have two genes for TOR. TOR signaling pathway operates in two functionally and structurally distinct complexes, TORC1 and TORC2^17^. TORC1 promotes translation, transcription of ribosomal protein genes, growth in response to sugar content and nitrogen availability but inhibits autophagy, while TORC2 governs cellular metabolism and reorganization of the cytoskeleton^18^.

In plants, several studies have deciphered TOR's crucial roles in developmental processes, including embryogenesis, seedling growth, root and shoot meristem activation, root hair elongation, chloroplast formation, photoautotrophic transition proliferation of leaf primordia, leaf expansion, flowering, and senescence to name few^19^. Inhibition of TOR causes quantitative changes in the phosphorylation status of hundreds of proteins in *Chlamydomonas reinhardtii* and *Arabidopsis thaliana*, indicating an indispensable role of this gene in regulating protein activity^20,21^. TOR activity is induced by glucose and sucrose by a complex mechanism^19,22^ and regulates meristem activity by inducing *E2F* and *WUSCHEL* transcription factors in root and shoot apical meristems, respectively^23,24^. Recent studies show TOR regulation of ribosomal protein gene activation and it also differentially regulates preinitiation complex formation at the promoters of the non-ribosomal protein genes thus, demonstrating a complex regulation of gene activation by TOR^25^. *TOR* overexpression lines of *A. thaliana* showed increased vulnerability to both bacterial and fungal pathogens, while plants with reduced TOR signaling exhibited increased resistance^26,27^. TOR antagonizes the actions of the classic defense hormones salicylic acid and jasmonic acid^28^. Abiotic and biotic stress affects plant growth and development by reprogramming transcriptional, translational, and metabolic pathways^27,29^. Previously, we have shown that TOR as an important regulatory factor for rhizobial infection and nodule development in *P. vulgaris* and hence essential for root nodule symbiosis^30^.

The nutritional status of the host plant is an important factor for successful AM fungal development^31^. Conditions that downregulate *TOR* expression could disrupt perception of the nutritional status of the plant and thus affect the symbiotic association. To study the function of legume TOR in AM symbiosis development and formation, we used RNA-interference to knockdown *TOR* transcript levels in transgenic hairy roots of common bean (*Phaseolus vulgaris*). We studied the symbiotic phenotype of the transgenic *PvTOR*-RNAi roots in detail and analyzed the spatiotemporal expression of *PvTOR* during AM symbiosis formation. Based on our results, we propose that *PvTOR* is required for fungal penetration, mycelial growth and arbuscule development in *P. vulgaris*.

## Results

### *PvTOR* is expressed during AM fungal symbiosis in wild-type *P. vulgaris*

**Figure 1:**
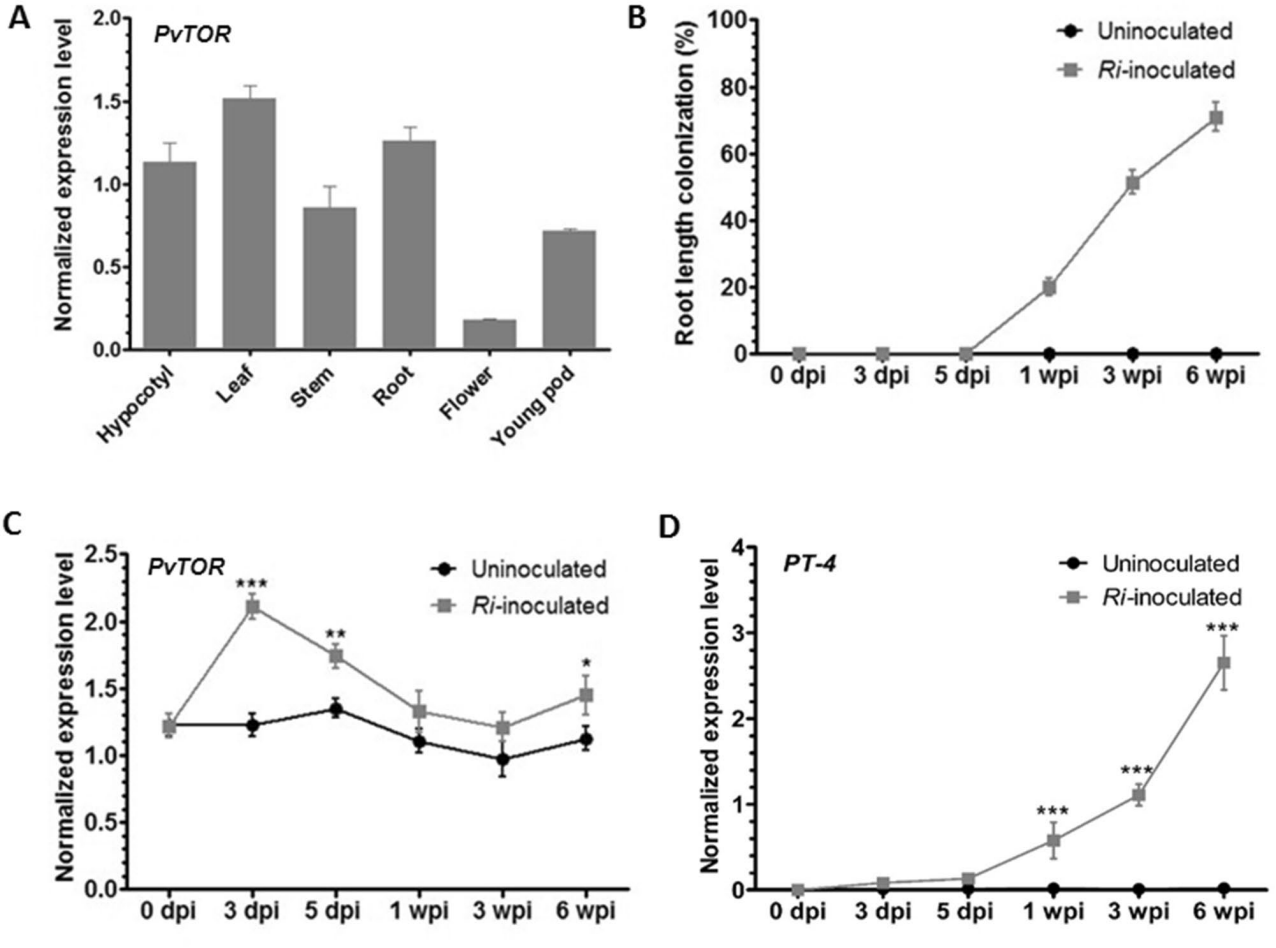
Expression of *PvTOR* in various tissues and in mycorrhized wild-type *P. vulgaris* plants. (**A**) RT-qPCR analysis of *PvTOR* transcript levels in different vegetative and reproductive organs. (**B**) AM fungal colonized roots were stained and assessed for percent mycorrhizal root length colonization under a light microscope at different time points. The remaining root portions were used for RT-qPCR analysis to measure (**C**) *PvTOR* and (**D**) *PT-4* expression in AM roots at different time points. Transcript accumulation was normalized to the expression of *EIF4a* and *IDE*, which were used as reference genes. For C and D, the statistical significance of differences between uninoculated and *Ri*-inoculated roots was determined using an unpaired two-tailed Student’s *t*-test (*P < 0.05; **P < 0.01; ***P < 0.001). For A–D, error bars refer to the SE of the mean of three biological replicates (n > 9). *Ri*, *R. irregularis*; dpi, days post inoculation; wpi, week(s) post inoculation.

To determine the role of *Pv*TOR in AM fungal symbiosis in the legume, *P. vulgaris*, we examined the temporal and spatial expression of *PvTOR* in wild-type *P. vulgaris*. Quantitative RT-PCR (RT-qPCR) analysis revealed that *PvTOR* expression varies among organs and tissues (Fig. 1A). *PvTOR* transcript levels were higher in vegetative organs (hypocotyl, leaf, stem, and root) than in reproductive organs (flower and young pod). To analyze *Pv*TOR expression during the AM fungal symbiosis, we inoculated 5-day-old wild-type seedlings with *Rhizophagus irregularis* under low Pi (10 µM phosphate (P)) conditions. Uninoculated plants grown under identical conditions were used as controls. Inoculated and uninoculated roots were harvested at various time points and a small portion of each sample was stained with trypan blue to measure the overall percentage of root length colonization (RLC)^32^ by AM fungi. All inoculated roots (1, 3, and 6 wpi [weeks post-inoculation] with *R. irregularis*) were colonized successfully, whereas the uninoculated control roots were free of such colonization (Fig. 1B).

We used the remaining portion of each root sample for RT-qPCR analysis of the expression levels of *PvTOR* and *PvPT-4*, a previously identified AM fungi-induced gene. The *PvTOR* mRNA levels were sharply increased at 3 dpi (days post inoculation) in inoculated roots and remained higher than in the uninoculated roots throughout the rest of the experiment (Fig. 1C). As expected, *PvPT-4* expression was induced only in inoculated roots in all tested samples (Fig. 1D). Together, these results indicate that *PvTOR* is expressed in a majority of *P. vulgaris* tissues and is upregulated under AM fungi symbiotic conditions.

### The tissue specificity of *PvTOR* expression changes in response to AM fungi

**Figure 2:**
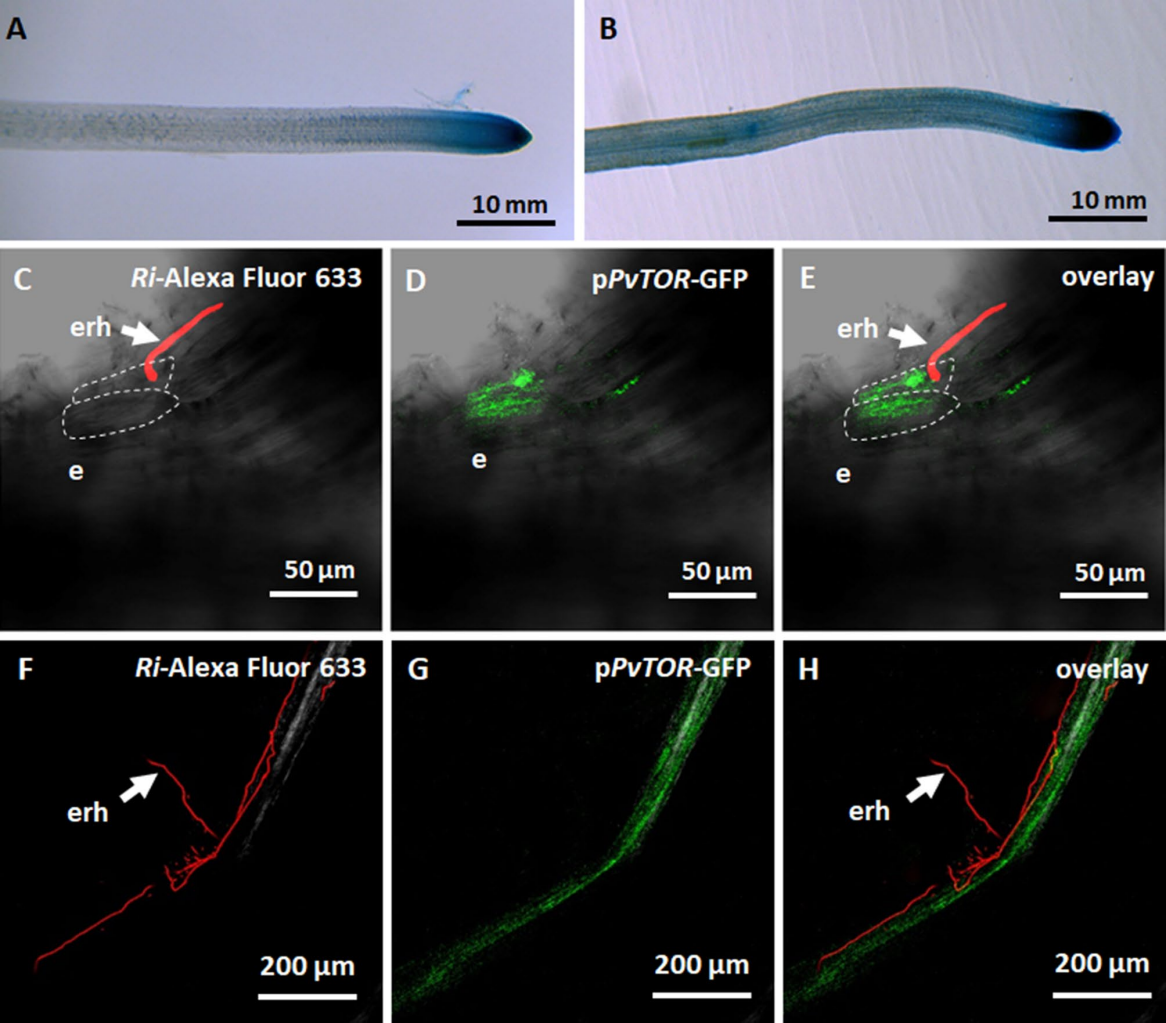
Spatial expression of the *PvTOR* promoter in *R. irregularis*-inoculated transgenic *P. vulgaris* roots. (**A–B**) Histochemical GUS staining of (**A**) uninoculated and (**B**) *R. irregularis-*inoculated *PvTOR*_*pro*_::*GUS*-*GFP* transgenic roots at 2 wpi. (**C–E**) Transmitted light and confocal images of *PvTOR*_*pro*_::*GUS*-*GFP* transgenic roots at 2 dpi with *R. irregularis* showing (**C**) extraradical hyphae in contact with root epidermis, (**D**) induction of *PvTOR*_*pro*_::*GUS*-*GFP* expression in the epidermal cells at the site of extraradical hyphal contact and (**E**) overlay image. (**F–H**) *PvTOR*_*pro*_::*GUS*-*GFP* transgenic roots at 2 wpi with *R. irregularis* showing (**F**) extraradical hyphae on the root epidermis surface, (**G**) induction of *PvTOR*_*pro*_::*GUS*-*GFP* expression in the root colonized by *R. irregularis*, and (**H**) overlay image. WGA Alexa Fluor 633 was used to stain (red) fungal cell walls. dpi, days post inoculation; wpi, week(s) post inoculation; e, epidermis; erh, extraradical hyphae.

The spatial expression patterns of *PvTOR* during AM fungal symbiosis were investigated using a promoter activity assay in transgenic roots. To create the reporter construct, the 1-kb region upstream of the translation start codon of *PvTOR* was fused to the chimeric reporter β-glucuronidase (*GUS*)-enhanced green fluorescent protein (*PvTOR*_*pro*_::*GUS*-*GFP*). The *PvTOR*_*pro*_::*GUS*-*GFP* reporter construct was transfected into *P. vulgaris* by hairy root transformation and the plants were then inoculated with AM fungi. In uninoculated transgenic roots, GUS staining showed that *PvTOR* was expressed primarily in the root tip (Fig. 2A). After inoculation with AM fungi, the promoter activity was enhanced in the root tip and could also be detected in the elongation and maturation zones of the root (Fig. 2B).

To further evaluate *PvTOR* promoter activity during AM fungal invasion, we used confocal microscopy to examine *PvTOR*_*pro*_::*GUS*-*GFP* transgenic roots. In this assay, *R. irregularis* was stained with wheat germ agglutinin (WGA) conjugated to Alexa Fluor 633, which produces far-red fluorescence. Once extraradical hyphae of the AM fungi came into contact with the root epidermis (Fig. 2C), *PvTOR* promoter activity (GFP fluorescence) was detected at the site of fungal penetration (Fig. 2D–E). *PvTOR* promoter activity was also observed along the mycorrhized root at 2 wpi (Fig. 2F–H). However, neither GUS staining nor GFP fluorescence was observed in uninoculated roots or in *R. irregularis* (data not shown). Taken together, these results show that the Pv*TOR* promoter was active during the course of AM symbiosis.

### *PvTOR* knockdown alters *P. vulgaris* root growth and the expression of root meristem regulatory genes

**Figure 3:**
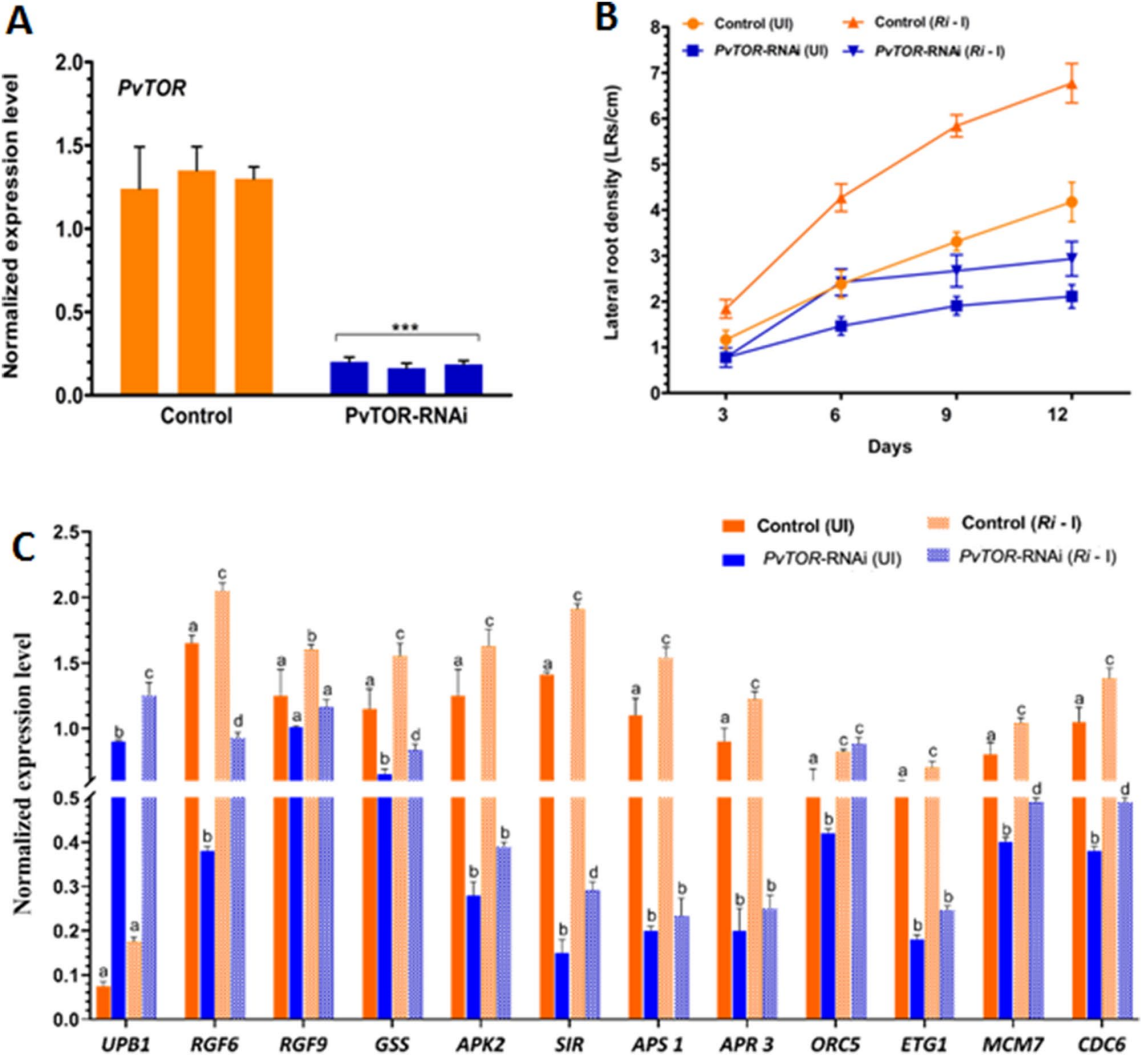
Knockdown of *PvTOR* inhibits AM fungus-stimulated lateral root production and downregulates the expression of root meristem regulatory genes in *P. vulgaris.* (**A**) Quantitative RT-PCR analysis of *PvTOR* transcript levels in control (empty vector) and *PvTOR*-RNAi roots at 10 days post emergence. Each bar represents three individual transformants (representative RNAi plant 1, 2, and 3). (**B**) Lateral root density in uninoculated and *Ri*-inoculated transgenic roots of control and *PvTOR*-RNAi at the indicated time points. (**C**) RT-qPCR analysis of the transcript levels of root meristem regulatory genes such as *UPB1*, *RGF6*, *RGF9*, *GSS*, *APK2*, *SIR*, *APS1*, *APR3*, *ORC5*, *ETG1*, *MCM7*, and *CDC6* in control and *PvTOR*-RNAi transgenic roots at 6 dpi with *Ri*. Quantitative RT-PCR was performed on cDNA of root meristem RNA samples. Transcript accumulation was normalized to the expression of *EIF4a* and *IDE*, which were used as reference genes. For A, an unpaired two-tailed Student’s *t*-test was used to assess statistical significance (***P < 0.001). For B, the statistical significance of differences was determined using Tukey’s test followed by two-way ANOVA the results were statistically significant at p < 0.05 except for the Control (UI) vs. *PvTOR*-RNAi (UI), Control (UI) vs. *PvTOR*-RNAi (Ri—I), *PvTOR*-RNAi (UI) vs. *PvTOR*-RNAi (Ri—I) at 3 day samples and Control (UI) vs. *PvTOR*-RNAi (Ri—I) at 6 day samples. For D, Tukey’s test followed by two-way ANOVA was used to asses statistical significance and the mean values of each gene with unlike letters were significantly different (P < 0.05). Error bars refer to the SD of the mean of three biological replicates (n > 9 for A & D, n > 30 for B). *Ri*, *R. irregularis.*

Myc-lipochitooligosaccharides produced by AM fungi during mycorrhizal colonization induce lateral root (LR) branching in *M. truncatula*^33^ and increase LR density in *P. vulgaris*^34^. In Arabidopsis, TOR specifically regulates the proliferation of primary root meristem cells^23^. To clarify the involvement of *Pv*TOR in AM fungi-induced changes in LR development, we used RNAi to knockdown *PvTOR* expression in transgenic hairy roots of *P. vulgaris*. Quantitative RT-PCR assays confirmed that *PvTOR* expression was reduced at least sixfold in the *PvTOR*-RNAi roots relative to control roots transformed with empty pTdT-DC-RNAi vector (Fig. 3A).

To analyze the effects of *PvTOR* knockdown on root growth, we measured the primary root growth and LR density of *PvTOR*-RNAi plants with and without inoculation with *R. irregularis*. We observed that the primary roots of *PvTOR*-RNAi plants were shorter than those of control plants at all observed time points, regardless of the presence or absence of *R. irregularis* (Fig. S1). In control plants, the LR density was much higher for inoculated roots than for uninoculated roots at all time points. By contrast, *PvTOR*-RNAi roots showed only a marginal increase in LR density at the early time points after inoculation (i.e., 6 dpi; Fig. 3B). Quantitative measurements of the root mass confirmed these observations; a marginal increase of LR mass was observed in inoculated *PvTOR*-RNAi roots relative to uninoculated *PvTOR*-RNAi roots and, in general, LR mass was significantly decreased in *PvTOR*-RNAi roots relative to control roots, whether inoculated or not (Fig. S2).

Next, we quantified the transcription of key genes involved in the TOR-mediated regulation of root meristem activity^23^. The transcription factor gene *UPB1*, whose overexpression inhibits root meristem expansion through redox control^35^, was transcriptionally upregulated in *PvTOR*-RNAi relative to control inoculated root meristems (Fig. 3C). Other genes were upregulated in inoculated roots relative to uninoculated roots, to levels significantly higher in control roots but only marginally higher in *PvTOR*-RNAi roots. These genes included those encoding *ROOT MERISTEM GROWTH FACTORS* (*RGF6* and *RGF9*), *SULFUR ASSIMILATION GLUTATHIONE SYNTHETASE* [*GSS*], *ADENOSINE-5'-PHOSPHOSULFATE KINASE 2* [*APK2*], *SULFITE REDUCTASE* [*SIR*], *ATP SULFURYLASE 1* [*APS 1*], and *APS REDUCTASE 3* [*APR 3*]), and *S-phase proteins E2F TARGET GENE 1* [*ETG1*], *MINICHROMOSOME MAINTENANCE 7* [*MCM7*], and *CELL DIVISION CONTROL 6* [*CDC6*], but not *ORIGIN RECOGNITION COMPLEX PROTEIN 5* [*ORC5*]) (Fig. 3 C).

In summary, knockdown of *PvTOR* inhibited primary root growth and LR formation in both uninoculated and *R. irregularis* inoculated roots. Similarly, the transcripts of genes encoding RGFs, sulfur assimilation, and S-phase proteins are suppressed in uninoculated and *R. irregularis* inoculated *PvTOR*-RNAi roots.

### Knockdown of *PvTOR* increases the length of AM extraradical hyphae

**Figure 4:**
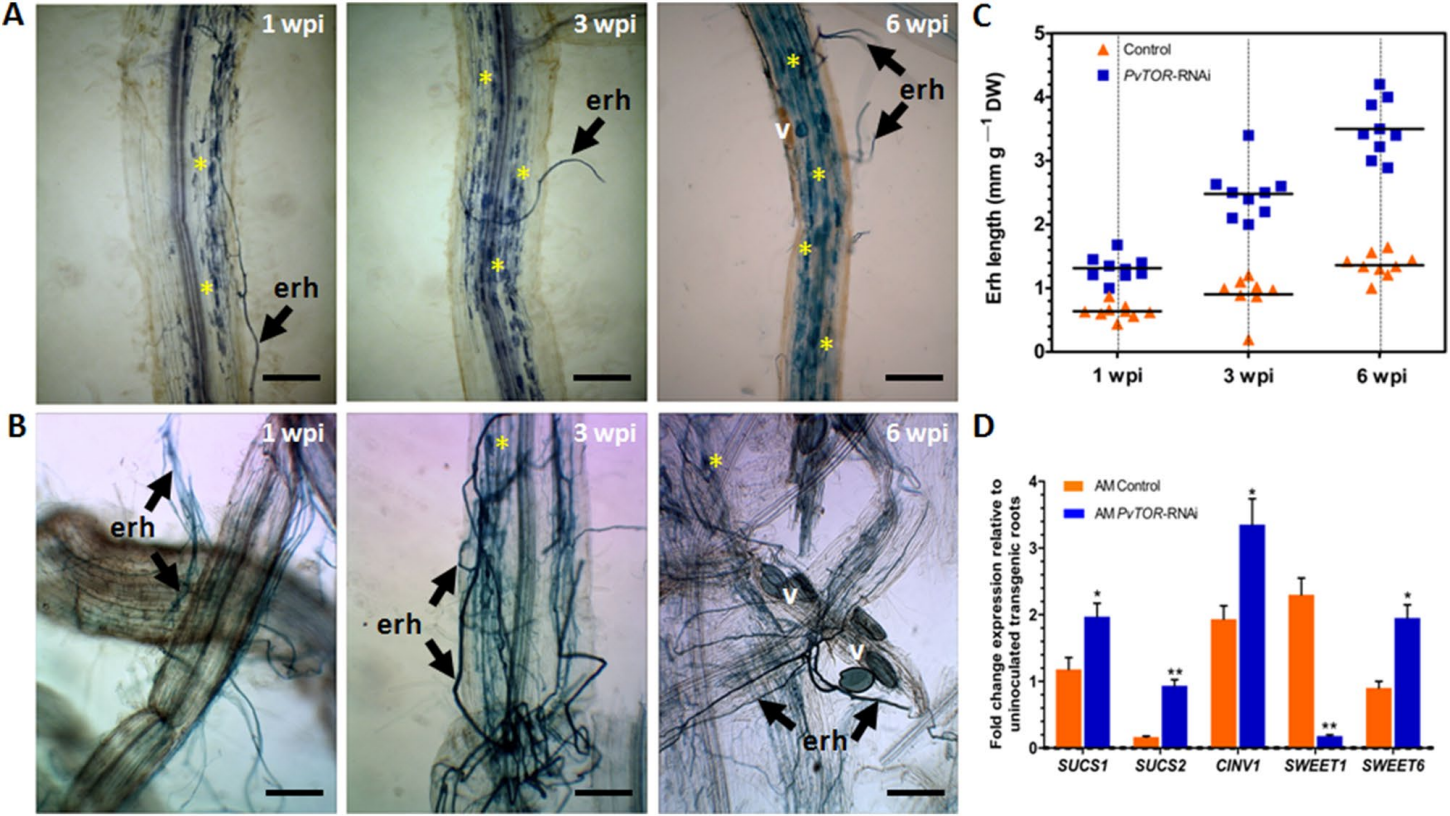
Arbuscular mycorrhizal extraradical hyphae in *P. vulgaris PvTOR*-RNAi roots. (**A–B**) Transgenic root surface showing extraradical hyphae of mycorrhiza in control (**A**) and *PvTOR*-RNAi (**B**) plants at 1, 3, and 6 week(s) post inoculation (wpi) with *R. irregularis*. Arbuscule containing cells are indicated with an asterisk. erh, extraradical hyphae; v, vesicle. Bars = 2 mm. (**C**) Length of arbuscular mycorrhizal extraradical hyphae relative to the dry weight of transgenic roots at 1, 3, and 6 wpi with *R. irregularis*. n = 9 plants per time point for each line (AM control and AM *PvTOR*-RNAi). (**D**) RT-qPCR analysis of the transcript levels of sugar metabolism and sucrose transport genes in control and *PvTOR*-RNAi transgenic roots at 1 wpi with *R. irregularis*. Data are the fold-change expression relative to uninoculated transgenic roots. Transcript accumulation was normalized to the expression of *EIF4a* and *IDE*, which were used as reference genes. The statistical significance of differences between AM control and AM *PvTOR*-RNAi roots was determined using an unpaired two-tailed Student’s *t*-test (*P < 0.05; **P < 0.01). Error bars refer to the SD of the mean of three biological replicates.

To examine the effect of *PvTOR* knockdown on the establishment of AM symbiosis, composite *P. vulgaris* plants (i.e., plants induced to form hairy roots by transformation with *Agrobacterium rhizogenes*) were inoculated with *R. irregularis* and monitored from 1 to 6 wpi. Light microscopy observations of inoculated root surfaces revealed that *PvTOR*-RNAi roots had more extensive extraradical hyphae (ERH) than control roots and that this difference increased over time (Fig. 4A,B).

**Figure 5:**
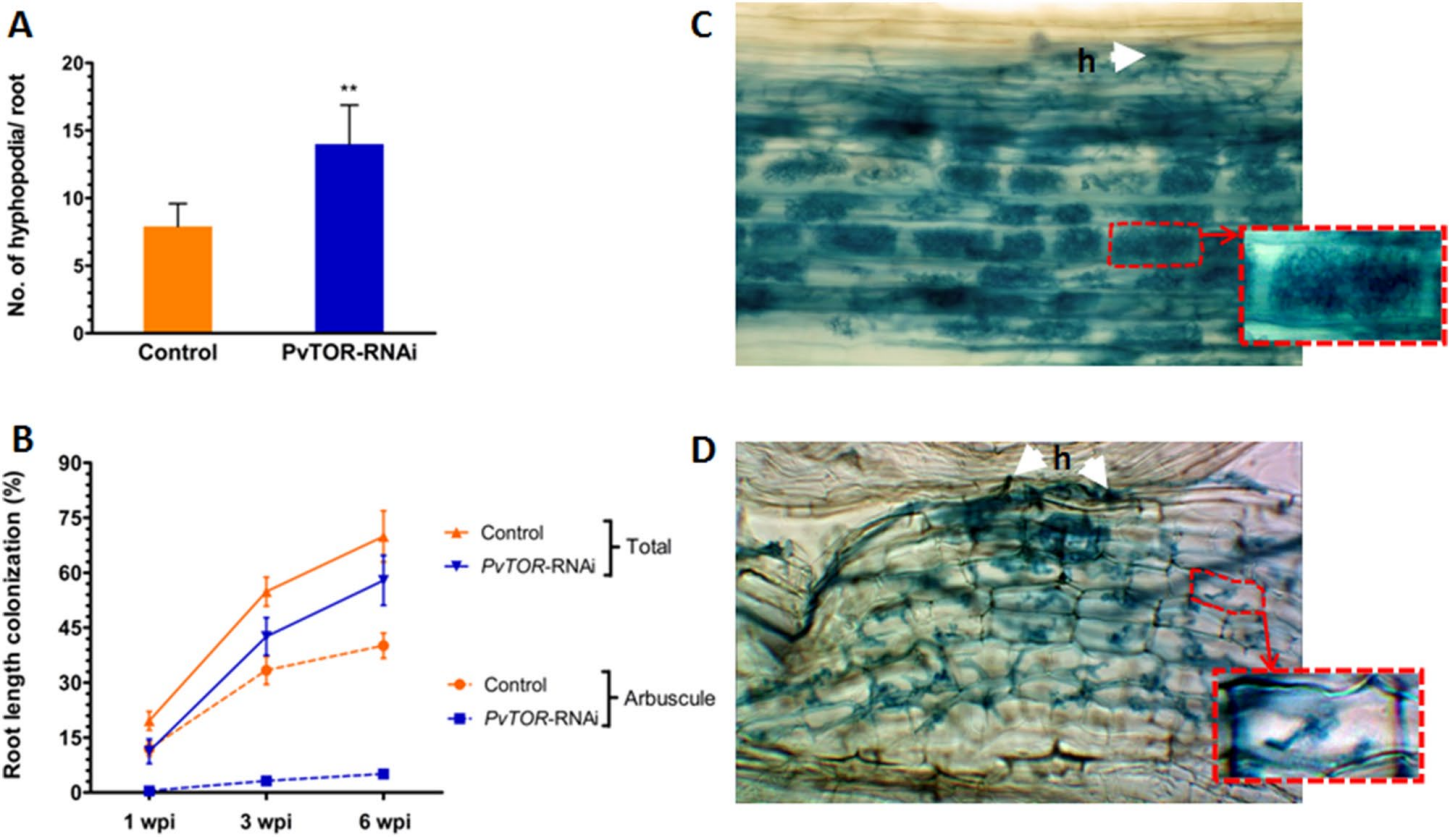
Quantification of hyphopodia and arbuscular mycorrhizal colonization in *P. vulgaris PvTOR*-RNAi roots. (**A**) Number of hyphopodia per transgenic root of control and *PvTOR*-RNAi bean plants at 6 wpi with *R. irregularis*. The statistical significance of differences between control and *PvTOR*-RNAi roots was determined using an unpaired two-tailed Student’s *t*-test (***P < 0.001). Error bars refer to the SD of the mean of three biological replicates (n > 30). (**B**) Total and arbuscular colonization levels in control and *PvTOR*-RNAi transgenic roots. The composite plants were harvested at 1, 3, and 6 wpi and mycorrhizal colonization parameters were quantified. Error bars refer to the SD of the mean of three biological replicates (n > 30). (C–D) Micrograph of *R. irregularis*-inoculated transgenic roots showing hyphopodia (arrowhead) and arbuscules in (**C**) empty vector (control) and (**D**) *PvTOR*-RNAi roots at 6 wpi. Insets show magnifications of an arbuscule containing cell (red-dashed regions). wpi, week(s) post inoculation; h, hyphopodia.

To further confirm this observation, we measured the lengths of the ERH. The mean length of ERH on *PvTOR*-RNAi roots was 2.5-fold greater than that of ERH on control roots at 6 wpi (Fig. 4C). It is worth noting that in *Medicago truncatula*, knockdown of AM symbiosis-induced *SUCROSE SYNTHASE 1* (*SUCS1*) impaired fungal colonization specifically, resulting in fewer radical hyphae and vesicles during AM symbiosis^36^. Further, *MtSWEET1b* is reported to be responsible for arbuscule maintenance by transporting sugar across the peri arbuscular membrane^37^. We identified the homologues of sugar transporter genes in *P. vulgaris* based on *M. truncatula* sequences. The phylogenetic alignment of the sequences showed that *MtSWEET1b* (Medtr3g089125), *PvSWEET1* (Phvul.009G134300) and *MtSWEET6* (Medtr3g080990), *PvSWEET6* (Phvul.006G000600) were grouped together in the same clade (Fig. S3). With the exception of *SWEET1*, the expression levels of sugar metabolism and sugar transport genes—namely, *SUCS1*, *SUCS2*, *CYTOSOLIC INVERTASE 1* (*CINV1*), and *SWEET6*-*related*— were found to be induced in AM fungi inoculated *PvTOR*-RNAi roots than in mycorrhized control roots (Fig. 4D). Subsequent observations on hyphopodia revealed that the longer ERH on *PvTOR*-RNAi roots were associated with a significantly higher number of hyphopodia compared to control roots (Fig. 5A, Fig. S4).

We further examined the effect of *Pv*TOR on AM fungal colonization by quantifying root length colonization (RLC) at various time points in control and *PvTOR*-RNAi roots. Total RLC, arbuscule RLC, and vesicle number were found to increase from 1 to 6 wpi in inoculated control roots. Inoculated *PvTOR*-RNAi roots showed significantly decreased total RLC (Fig. 5B) and significantly increased vesicle numbers relative to the control at all tested time points (Fig. S5). Furthermore, arbuscule RLC was more than eightfold lower in *PvTOR*-RNAi roots than in the control (Fig. 5B). Interestingly, the majority of arbuscules present in *PvTOR*-RNAi roots were abnormal (Fig. 5C–D). These results suggest that *PvTOR* knockdown causes an increase in ERH, hyphopodia, and vesicles but a decrease in total RLC and arbuscule RLC.

### *Pv*TOR is indispensable for arbuscule maturation

**Figure 6:**
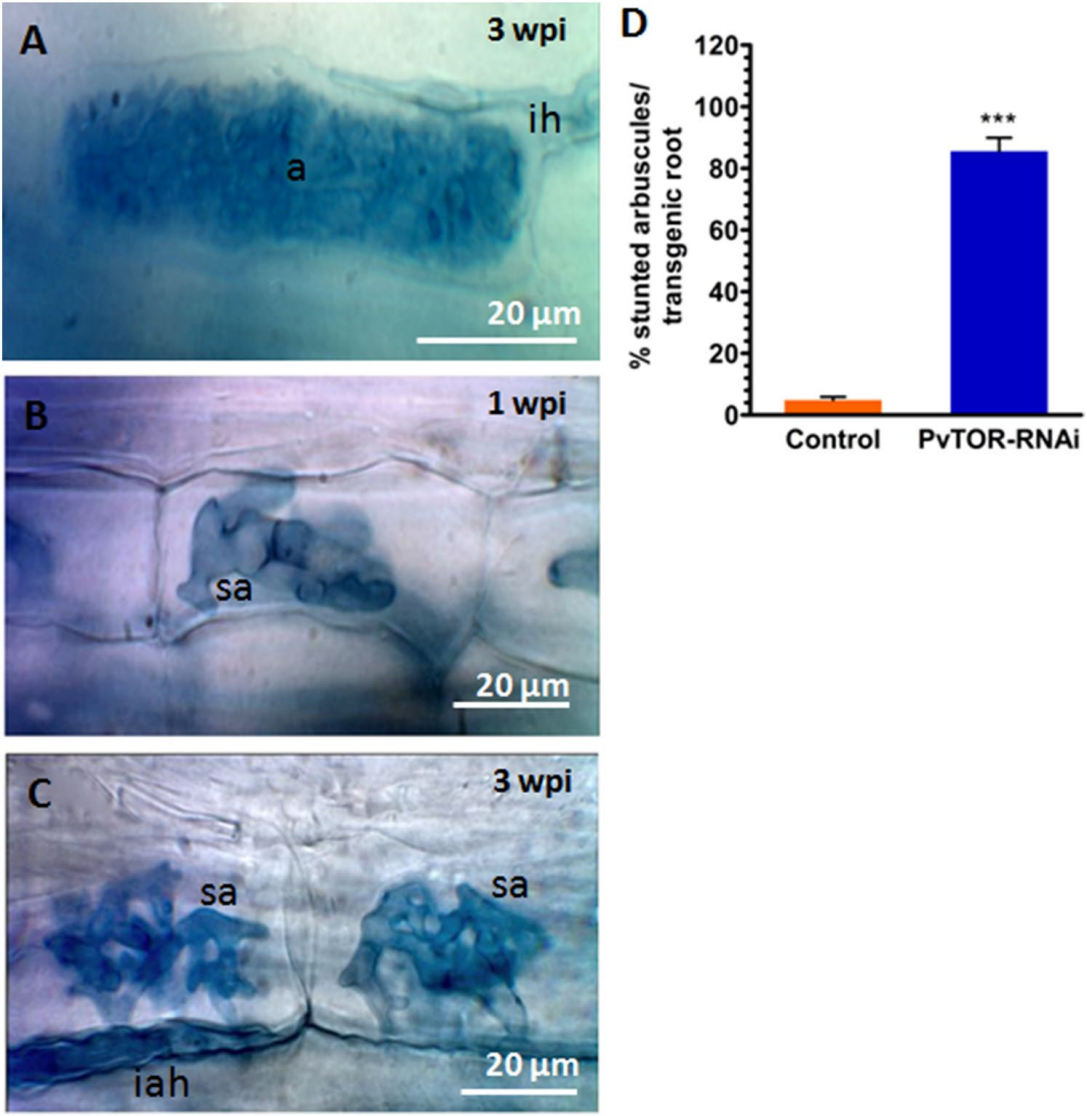
Arbuscular mycorrhizal phenotype of *P. vulgaris PvTOR*-RNAi roots colonized by *R. irregularis*. (**A**) The arbuscules in the cortex cells of the control transgenic roots are well developed. (**B–C**) Stunted arbuscules in the cortex cells of the *PvTOR*-RNAi transgenic roots at 1 wpi and 3 wpi, respectively. (**D**) Quantification of stunted arbuscules per transgenic control and *PvTOR*-RNAi roots at 3 wpi. The statistical significance of differences between control and *PvTOR*-RNAi roots was determined using an unpaired two-tailed Student’s *t*-test (***P < 0.001). Error bars refer to the SD of the mean of three biological replicates (n > 30 for D). a, arbuscule; ih, intercellular hyphae; sa, stunted arbuscule, iah, intracellular hyphae; wpi, week(s) post inoculation, Ri, *R. irregularis*.

**Figure 7:**
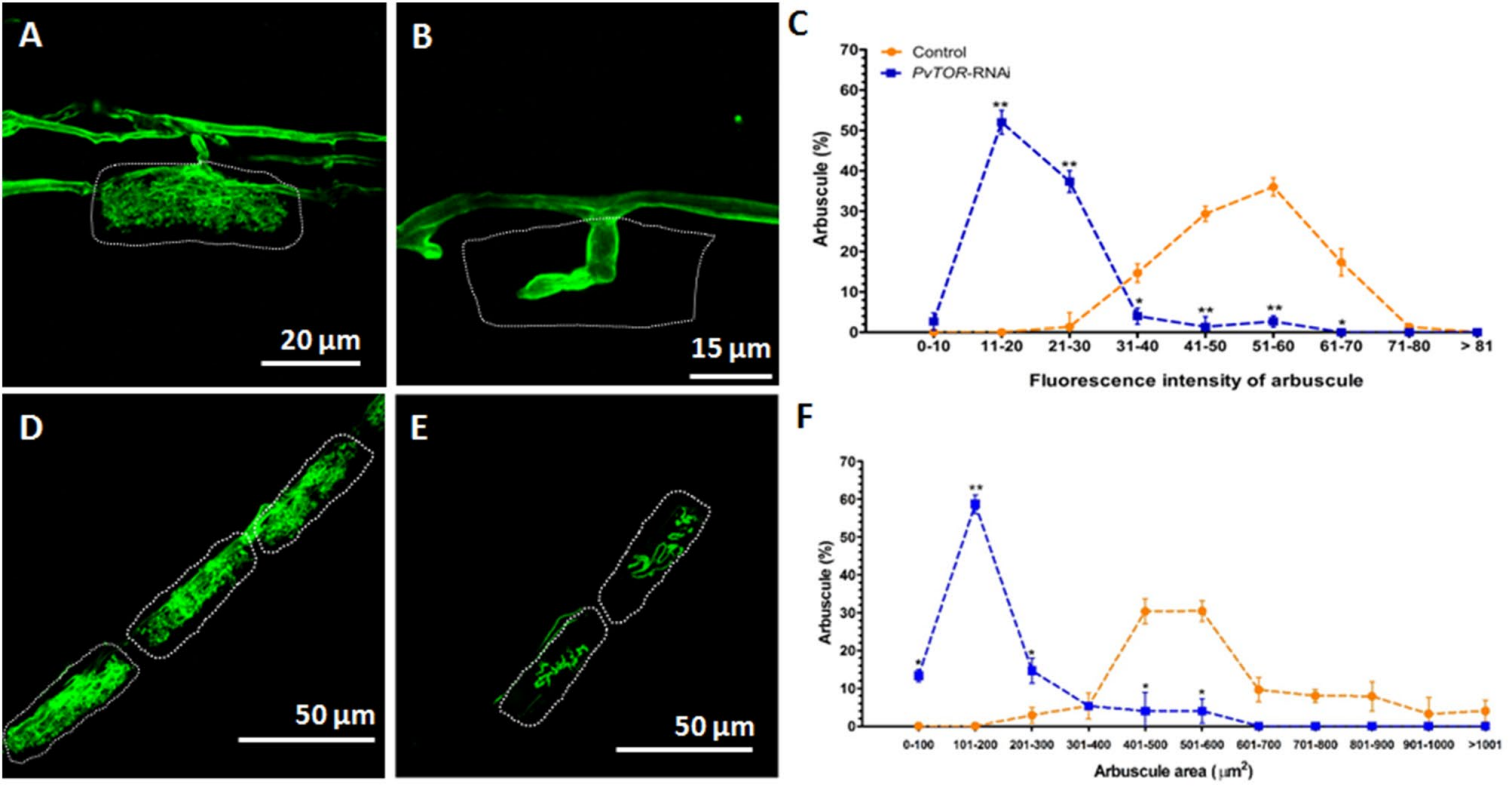
Analysis of arbuscule size in *P. vulgaris PvTOR*-RNAi roots colonized by *R. irregularis*. (**A-B**) Confocal images of *R. irregularis* arbuscules stained (green) with wheatgerm agglutinin (WGA) conjugated with Alexa Fluor 488 in the cortex cells of control (**A**) and *PvTOR*-RNAi (**B**) bean plants. (**C**) Frequency of the fluorescence pixel intensity in arbuscule populations measured in control and *PvTOR*-RNAi transgenic roots at 3 weeks post inoculation. (D–E) Confocal images of arbuscules stained with WGA conjugated with Alexa Fluor 488 in the cortex cells of control (**D**) and *PvTOR*-RNAi (**E**) bean plants. Dotted lines mark the arbuscule area. (**F**) Frequency of the distribution of the arbuscule area in arbuscule populations measured in control and *PvTOR*-RNAi transgenic roots. For C and F, mean values of 25 arbuscules per composite plant and a total of three plants are shown. The statistical significance of differences between control and *PvTOR*-RNAi roots was determined using an unpaired two-tailed Student’s *t*-test (*P < 0.05; **P < 0.01). Error bars refer to the SE of the mean.

Given that *PvTOR* knockdown affected AM-symbiosis, we examined this phenotype in more detail. We inspected control and *PvTOR*-RNAi roots for fungal structures at 1 and 3 wpi with *R. irregularis*. Closer observation revealed that during the initial phases of symbiosis, such as hyphopodia formation and fungal entry into the cortex (intraradical hyphae; IRH), occurred normally in *PvTOR*-RNAi roots (Fig. 5C–D; Fig. S4). By contrast, although arbuscule development was initiated in *PvTOR*-RNAi roots, the arbuscules were stunted and clumped (Fig. 6B–C). Furthermore, the *PvTOR*-RNAi arbuscules were smaller and less densely branched (Fig. 7B, E) than those of the controls (Fig. 6A, 7A, D).

As another measure of arbuscule development, roots were stained with WGA conjugated with Alexa Fluor 488 to label fungal structures and the fluorescence intensity of arbuscule populations was measured using pixel intensity. The majority of arbuscules in *PvTOR*-RNAi transgenic roots showed fluorescence intensities ranging from 11–30 a.u. compared to intensities of 30–70 a.u. in controls (Fig. 7C). To precisely determine the arbuscule size range in control and *PvTOR*-RNAi roots, we measured the cross-sectional areas of arbuscule populations and sorted them into size categories. The majority of arbuscules in control roots had an area of 400–600 µm^2^, while in *PvTOR*-RNAi roots the area was significantly reduced, with most of arbuscules in the 100–200 µm^2^ range (Fig. 7F). Over 90% of arbuscules on *PvTOR*-RNAi roots were stunted and this phenotype persisted at all observed time points (Fig. 6D; Fig. S6). By contrast, the control arbuscules were well developed and highly branched, completely filling the arbuscule-containing cortical cells (Fig. 6A, 7A,D). Interestingly, the number of AM vesicles was significantly higher in the *PvTOR*-RNAi roots than in the control (Fig. S5).

To test whether *PvTOR* is required for the uptake of P by the AM fungi, we measured P concentrations in inoculated and uninoculated *PvTOR*-RNAi and control roots at 3 and 6 wpi. A portion of the root samples was stained to determine AM fungal colonization and the total % RLC was approximately the same as previously shown in Fig. 5B. However, in inoculated plants, the total P concentrations were significantly lower in *PvTOR*-RNAi roots i.e., 40.2% at 3wpi and 46.9% at 6wpi; and 53.2% at 3wpi and 61.7% at 6wpi in shoots relative to the controls. The same was true of uninoculated plants; P concentrations were lower in *PvTOR*-RNAi roots and shoots than in the corresponding control samples (Fig. S7), confirming that in the absence of *PvTOR*, P uptake and transport are disturbed. Therefore, we conclude that *PvTOR* is essential for mycorrhizal P uptake in *P. vulgaris*.

### Activation of the common symbiosis pathway and expression of AM fungi-induced genes are altered in *PvTOR*-knockdown roots

**Figure 8:**
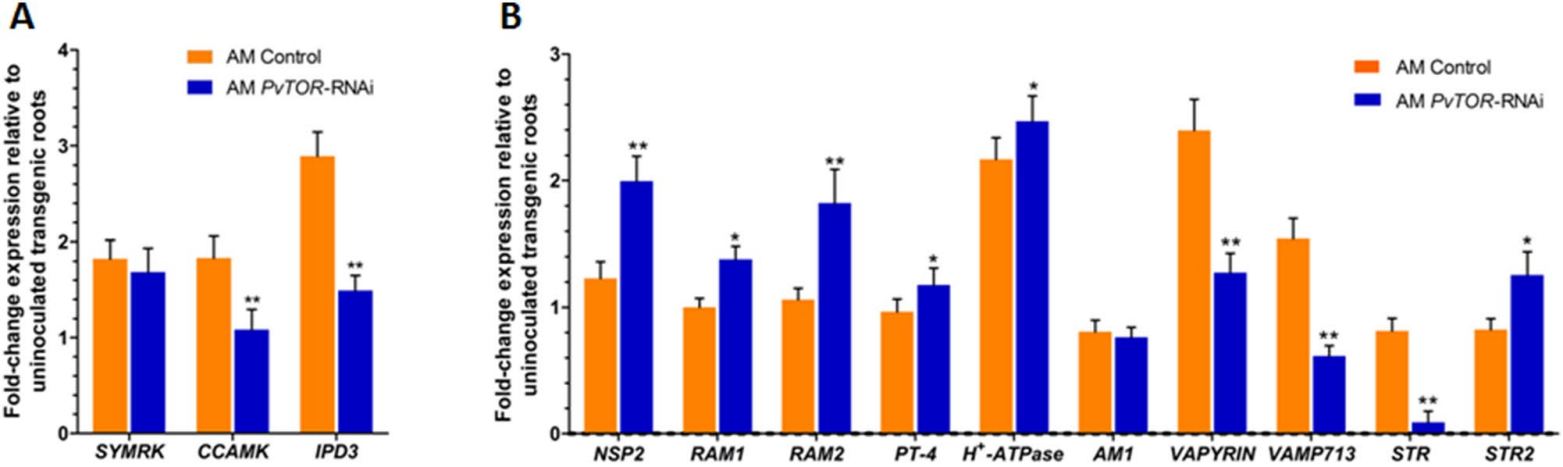
Expression of *P. vulgaris* AM regulatory genes in *R. irregularis*-inoculated transgenic roots. (**A**) RT-qPCR analysis of the transcript levels of CSSP genes and AM fungi induced TFs genes in control and *PvTOR*-RNAi transgenic roots at 1 wpi. (**B**) RT-qPCR analysis of the transcript levels of mycorrhizal-induced genes in control and *PvTOR*-RNAi transgenic roots at 1 wpi. (**A** & **B**) Quantitative RT-PCR was performed on cDNA of root RNA samples. The statistical significance of differences between mycorrhized roots of control and *PvTOR*-RNAi plants was determined using an unpaired two-tailed Student’s *t*-test (*P < 0.05; **P < 0.01). Error bars refer to the SE of the mean of three biological replicates (n > 9). CSSP, common symbiotic signaling pathway; AM, arbuscular mycorrhiza; TFs, transcription factors; wpi, week post inoculation.

In *M. truncatula*, induction of *GRAS*-type transcription factors specific to mycorrhizal signaling depends on activation of the common symbiosis signaling pathway (CSSP)^38^. To confirm that this is also the case for *R. irregularis*-inoculated transgenic roots of *P. vulgaris*, we used RT-qPCR to analyze transcript accumulation of the *P. vulgaris* CSSP genes, *SYMRK* (Phvul.002G143400), *CCAMK* (Phvul.011G186900), and *IPD3* (Phvul.002G128600), and of genes encoding the AM symbiosis-specific *GRAS*-type TFs, *NODULATION SIGNALING PATHWAY 2* (*NSP2*) (Phvul.008G165200) *REDUCED ARBUSCULAR MYCORRHIZATION 1* (*RAM1*) (Phvul.001G089900) and *RAM2* a *GLYCEROL-3-PHOSPHATE ACYL TRANSFERASE* (*GPAT*) (Phvul.007G233600). Transcripts for both *CCAMK* and *IPD3* were less abundant in *PvTOR*-RNAi roots, while there was no difference in *SYMRK* expression relative to controls (Fig. 8A). Transcripts for *RAM1* and *RAM2* were significantly more abundant in *PvTOR*-RNAi roots than in the control. AM specific markers *PvPT4* and *P. vulgaris* homologues Phvul.003G143400 and Phvul.010G050900 of *Oryza sativa AM1*^39^ and *M. truncatula H*^+^*ATPase*, *HA1*^40^ were found to be induced in *PvTOR*-RNAi roots confirming successful colonization of the symbiont (Fig. 8B).

Other genes induced by AM symbiosis include *VAPYRIN* (*VPY*)^41^ and *VAMP* (*SNARE*)^42^ in *M. truncatula* and *STR1* and *STR2* in *Oryza sativa*^43,44^. Mutation of these genes limits arbuscule growth of the AM fungi, resulting in a small and stunted arbuscule phenotype^43,44^. We measured the expression of the *P. vulgaris* homologs of these genes in inoculated roots and found that—with the exception of *STR2*—their transcript levels were significantly lower in *PvTOR*-RNAi roots than in control roots (Fig. 8B). Together, these results suggest that knockdown of *PvTOR* disrupts the expression of genes involved in arbuscule development but not those encoding AM symbiosis-specific *GRAS*-type TFs.

## Discussion

In plants, TOR plays a central regulatory role in modulating multiple cellular activities, including embryogenesis, meristem activation, root and leaf growth, flowering, and life span determination, as well as controlling photosynthesis, autophagy, and senescence^23,45,46^. Information about the role of this important protein in the regulation of symbiotic interactions is quite fragmentary. We previously showed that in the model legume *P. vulgaris*, *TOR* is involved in regulating rhizobial symbiosis, including infection thread progression and nodule organogenesis^30^. The focus of our current study was to improve our understanding of the role of *Pv*TOR in regulating interactions with another important endosymbiont, AM fungi. To this end, we analyzed the expression pattern of *PvTOR* in *P. vulgaris*, in the presence and absence of AM fungi, and studied the effect of *PvTOR* knockdown on AM symbiosis.

In terms of *PvTOR* expression, our key finding was that *PvTOR* promoter activity, assayed in *P. vulgaris* hairy roots transformed with a *PvTOR*_*pro*_::*GUS*-*GFP* reporter construct, increased in response to AM inoculation. The promoter activity in root cortical cells increased in coordination with the ramifying AM fungal mycelia, from the hyphopodium to the ERH to the IRH, an indication of mycorrhiza specific *PvTOR* promoter expression.

Transformation of *P. vulgaris* hairy roots with a pTdT-*PvTOR* RNAi construct resulted in an almost six-fold reduction in *PvTOR* transcript abundance. This *PvTOR* knockdown inhibited the proliferation of lateral roots that is normally caused by AM inoculation^47^. The *TOR* signaling pathway is also known to regulate the expression of genes involved in the proliferation of progenitor cells for root meristem activation and growth in Arabidopsis^23,45^. An analysis of the expression of root meristem regulatory genes (RGF, sulfur assimilation, and S-phase genes) in uninoculated *PvTOR*-RNAi hairy roots revealed significantly lower transcript levels relative to *PvTOR*-RNAi hairy roots inoculated with AM fungi at 6 dpi. We also observed increased expression of the transcription factor gene *UPB1,* whose overexpression is known to inhibit root meristem expansion through redox control^35^. We propose that *UPB1* is one of the genes regulated by *Pv*TOR, though further experimental studies are required to verify how TOR and redox regulatory signaling execute the cell proliferation through UPB1 transcription factor.

The ERH perform two main functions: they increase the surface area for mineral and water absorption from the soil and transport to the host via the arbuscule-cortical cell interface and they provide structures capable of colonizing new roots via hyphopodia^48^. Our observation shows that ERH became more extensive when the arbuscules were truncated by *PvTOR* knockdown.

AM fungal development in the host is governed by the nutritional status of the host plant. In response to AM colonization, the sink strength of host plant roots increases, allowing more sucrose to be unloaded from the phloem and exported toward the arbuscular cells. This increase in sink strength involves the activity of sucrose-cleaving enzyme invertases (*INV*) and *SUCROSE SYNTHASES* (*SUCS*) and the tight regulation of sucrose transporters^49,50^. AM-inoculated *PvTOR*-RNAi roots showed high expression levels of the sucrose synthase genes *SUCS1* and *SUCS2* and the *CYTOSOLIC INVERTASE* gene *CINV1.* The most important finding in these tissues was the transcript downregulation of the sugar transporter *PvSWEET1*, a homologue of *MtSWEET1b*. In *M. truncatula*, *SWEET1b* is localized to the periarbuscular membrane and is responsible for providing the arbuscule containing cells with the required carbon source for the growth and differentiation of arbuscules. However, the gene encoding the sugar transporter *PvSWEET6* was induced in AM-inoculated *PvTOR*-RNAi roots by two-fold compared to AM-inoculated controls. Hence, *PvSWEET6* could be the potential sugar transporter involved in the AM-specific source-to-sink sucrose transport in *PvTOR*-RNAi plants. Due to the stunted nature of arbuscules in *PvTOR*-RNAi roots, the available carbon source might be taken up by intraradicular hyphae via an unknown mechanism, as previously proposed by Bago and colleagues^51^. The increase in vesicle numbers could be to store the extra carbon received from the host.

An increase in ERH was also found to increase the number of hyphopodia. The *GRAS*-type transcription factors *RAM1*^52^ and *RAM2*^53^ function in hyphopodia formation. Similarly, in *P. vulgaris*, there was an increase in transcript accumulation of *NSP2*, *RAM1*, and *RAM2* in mycorrhized *PvTOR*-RNAi roots, providing molecular confirmation for the increased hyphopodia phenotype.

Analyses of the stunted arbuscule phenotype of *PvTOR*-RNAi roots revealed that the expression levels of *VPY*^41,54^, *VAMP*^55^, and *STR*^43,44^ were supressed. *STR* and *STR2* function as a heterodimer in the periarbuscular membrane that may possibly export a nutrient signal molecule essential for arbuscule development, as in *M. truncatula*^43^. In *PvTOR*-RNAi roots, *STR* expression was significantly reduced whereas *STR2* expression increased during AM symbiosis, implying that in the absence of STR, STR/STR2 heterodimer complex formation is affected and could account for the stunted arbuscules in *PvTOR*-RNAi roots.

Taken together, our study suggests that *PvTOR* permits arbuscule maturation during AM symbiosis in *P. vulgaris* by modulating AM-specific sugar transporters and arbuscule-specific genes. Though there was a surge in mycelial growth and hypopodial numbers, the P content of the *PvTOR*-RNAi plants did not differ from that of the controls. To improve our understanding of the involvement of *Pv*TOR in the common symbiotic signaling pathway of legumes, future studies should examine the downstream proteins, subcellular localization of TOR, and physiological mechanism of legume TOR regulation during symbiotic interactions.

## Methods

### Plant materials, inoculation, and growth conditions

Seeds of *Phaseolus vulgaris* L. cv. Negro Jamapa were obtained from Instituto de Biotecnología, UNAM, Mexico. The seeds were surface-sterilized^56^, germinated in the dark on wet filter paper for two days at 28 °C, transferred to sterile vermiculite, and grown under a 16-h photoperiod at 28 ± 1 °C. All the experiments involving plants were carried out in accordance with appropriate guidelines. Five-day-old plants were inoculated with *Rhizophagus irregularis* (800 spores/plant) [Symplanta, Darmstadt, Germany] and irrigated twice weekly with half-strength B&D solution^57^ containing a low concentration of potassium phosphate (10 µM, K_2_HPO_4_) to promote AM colonization^13^. At different time points, a portion of root sample (50% of total root volume) was excised from each plant, immediately frozen in liquid nitrogen, and stored at –80 °C for RNA extraction. The remaining portion of the root samples was stained to determine the percent root length colonization of AM fungi^32^. A set of plants grown separately under identical conditions but without *R. irregularis* inoculation served as controls.

### Plasmid construction and generation of composite plants

To develop the RNAi construct of *PvTOR*, a fragment corresponding to the non-conserved region of the C terminus and 3′-UTR of *PvTOR* (Phvul.002G049900) was amplified from the cDNA isolated from the root tips of 2-day-old germinated *P. vulgaris*, using specific oligonucleotides (Table S1). The PCR product was recombined with pTdT-DC-RNAi vector using the Gateway system (Invitrogen, Carlsbad, California, USA). The correct orientation of the clone was confirmed by sequencing the insert of the plasmid. Empty pTdT-DC-RNAi vector was used as the control.

Upstream of the *TOR* translation start site, a 1-kb promoter fragment was amplified from *P. vulgaris* genomic DNA using specific primers (Table S1) and cloned into the pENTR/SD/D-TOPO vector (Invitrogen, Carlsbad, California, USA). The Gateway LR reaction was performed between the entry vector pENTR/SD/D-TOPO-*PvTOR* and the destination vector pBGWSF7.0 according to the manufacturer’s instructions (Invitrogen). The *Agrobacterium rhizogenes*/K599 strain carrying the corresponding constructs was used to initiate hairy root formation on *P. vulgaris* tissues and form composite plants after transformation^56^. Transgenic hairy roots expressing the *PvTOR*-RNAi vector or *PvTOR*_*pro*_::*GFP*-*GUS* were selected under an epifluorescence stereomicroscope based on red fluorescent protein (RFP) and green fluorescent protein (GFP) expression, respectively. RFP fluorescence was excited at 561 nm by a solid-state laser and emission was filtered using a band pass filter of 640/50 nm. GFP fluorescence was excited with a blue argon ion laser (488 nm) and emitted fluorescence was collected from 510 to 540 nm.

### *PvTOR* promoter analysis

The *PvTOR*_*pro*_::*GFP*-*GUS* promoter construct was transfected into common bean cv. Negro Jamapa by hairy root transformation, and the resulting transgenic composite plants were inoculated with *R. irregularis* (~ 800 spores per plant). The roots were harvested at 5–14 dpi and stained either for promoter fusion GUS activity according to Jefferson^58^ or WGA (Wheat Germ Agglutinin) conjugated to Alexa Fluor 633 (Invitrogen, Carlsbad, California, USA) to visualize fungal structures^59^ in RED fluorescence, using a ZEISS LSM-510 confocal laser-scanning microscope. The *PvTOR* promoter activity was monitored in transgenic roots expressing *PvTOR*_*pro*_::*GUS*-*GFP*. WGA-Alexa Fluor 633 (red channel) was excited with an argon ion laser (633 nm), and emitted fluorescence was collected from 652 to 752 nm.

### RNA extraction and quantitative real-time PCR analysis

Total RNA was isolated from *P. vulgaris* roots using TRIzol reagent, according to the manufacturer’s recommendations (Thermo Scientific, Waltham, USA). Genomic DNA contamination from RNA samples was eliminated by incubating the samples with RNase-free DNase (1 U µl^–1^) at 37 °C for 15 min and then at 65 °C for 10 min. RNA integrity and concentration were determined by electrophoresis and NanoDrop ND-2000 (Thermo Scientific, Wilmington, USA) spectrophotometry, respectively.

Quantitative real-time PCR was performed using an iScript One-step RT-PCR Kit with SYBR Green (Bio-Rad, Hercules, California, USA), following the manufacturer’s instructions, in an iQ5 Multicolor Real-time PCR Detection System (Bio-Rad, Hercules, California, USA). Each reaction was set up using 40 ng of RNA as template. A control sample, which lacked reverse transcriptase (RT), was included to confirm the absence of contaminant DNA. Relative gene expression levels were calculated using the formula 2^–ΔCT^, where cycle threshold value (ΔCT) is the CT of the gene of interest minus the CT of the reference gene. *P. vulgaris EIF4a* (Phvul.010G136300) and *IDE* were used as control genes, as previously described^23,60^. The relative expression values, normalized with two reference genes, were calculated according to Vandesompele and colleagues^61^. The data are averages of three biological replicates and each sample was assessed in triplicate. The expression of genes listed in Table S1 was quantified using gene-specific oligonucleotides.

### Root growth parameters

Composite plants grown in pots of vermiculite and irrigated with B&D medium were used to determine root growth parameters and superoxide accumulation in transgenic roots. Transgenic roots expressing RFP were selected at various intervals from both uninoculated and *R. irregularis*-inoculated plants and root growth parameters were recorded. Lateral root density was calculated using the formula: D = LR/L´, where D = density of lateral roots; LR = number of lateral roots; and L´ = length of the main root between the first and last lateral root^62^.

### Quantification of mycorrhizal colonization and microscopy analysis

The AM fungi-inoculated roots were sampled at 1, 3, and 6 wpi and stained with trypan blue using the modified histochemical staining method^32^ or WGA-Alexa Fluor 488^59^ to measure the mycorrhizal colonization. Using a light microscope (DMLB Bright-field Microscope; Leica, Wetzlar, Germany), trypan blue-stained root samples were analyzed to visualize fungal structures (extraradical hyphae, hyphopodia, intraradical hyphae, vesicles, and arbuscules) and assess the root length colonization (percent RLC) as per McGonigle and colleagues^32^. Lengths of extra-radical hyphae (ERH) were determined according to a published protocol^63^ with some modifications^64^. Arbuscule size was measured using images obtained using a ZEISS LSM-510 confocal laser scanning microscope (ZEISS, Oberkochen, Germany). Z-stacks of Alexa Fluor 488-stained mycorrhized roots were generated from 12–18 serial images taken at increments of 1.25 µM, and analyzed using the LSM 5 tool. Alexa Fluor 488 (green channel) was excited with an argon ion laser (488 nm) and emitted fluorescence was collected from 510 to 540 nm.

### Quantification of phosphorus in leaves

To estimate total phosphorus levels in the leaves of composite plants, the dried leaf powder was wet digested using the nitric-perchloric acid method^65^ and the digested samples were dissolved at 1:20 (w/v) in distilled water and quantified spectrophotometrically (optical density 470 nm). The standard curve was prepared^65^ using the phosphorous containing salt, KH_2_PO_4_.

### Statistical analysis

Statistical analysis was performed using Prism Mac 6 (GraphPad Software, California, USA). An unpaired two-tailed Student’s *t*-test or two-way ANOVA was used to determine the statistical significance of differences between different groups. Single, double, and triple asterisks indicate differences that are statistically significant (*P* < 0.05 or *P* < 0.01) or highly significant (*P* < 0.001), respectively.

## Supplementary Information

Supplementary Information.
