# Supplementary material for: Target of rapamycin, PvTOR, is a key regulator of arbuscule development during mycorrhizal symbiosis in Phaseolus
Source: Sci Rep. 2021 May 31;11:11319. doi: 10.1038/s41598-021-90288-2 (PMC8166948; doi:10.1038/s41598-021-90288-2)
Supplement: Supplementary file 1 — Supplementary Information. [file 41598_2021_90288_MOESM1_ESM.pdf]

Target of Rapamycin, *PvTOR*, Is a Key Regulator of Arbuscule Development during Mycorrhizal Symbiosis in *Phaseolus*

Manoj-Kumar Arthikala[1#], Kalpana Nanjareddy[1], Lourdes Blanco[2], Xóchitl Alvarado-Affantranger[3], Miguel Lara[2]

[1]Ciencias Agrogenómicas, Escuela Nacional de Estudios Superiores Unidad León-Universidad Nacional Autónoma de México (UNAM), León, C.P. 37684, Guanajuato, México.

[2]Departamento de Biología Molecular de Plantas, [3]Laboratorio Nacional de Microscopía Avanzada, Instituto de Biotecnología, UNAM, C.P. 62271 Cuernavaca, México.

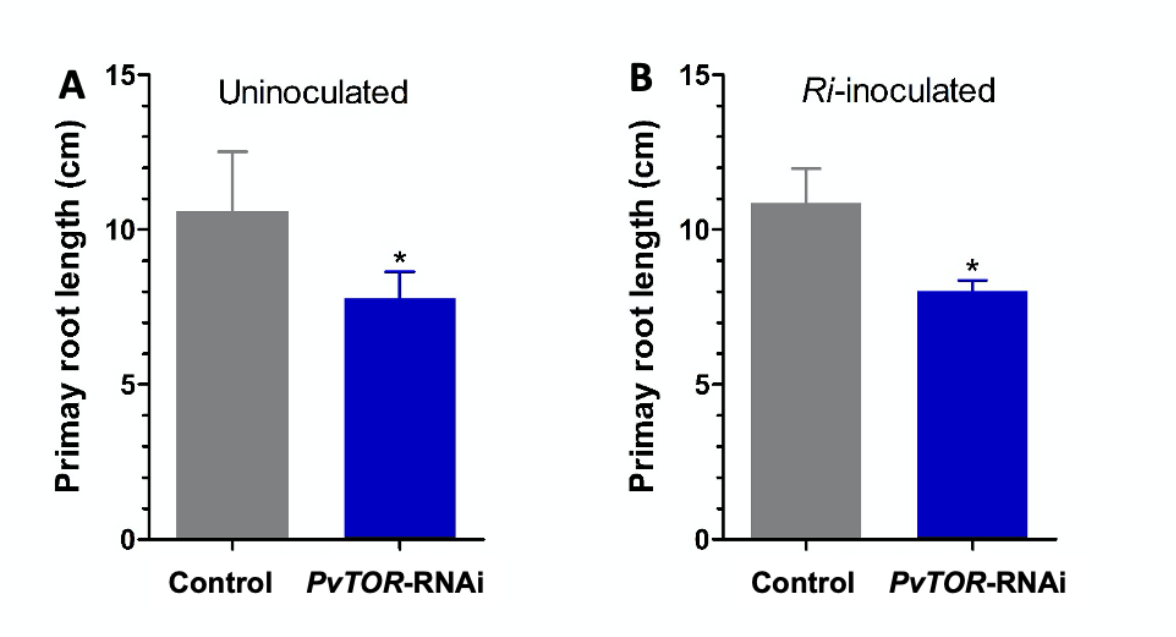

Figure S1. Knockdown of *PvTOR* inhibits primary root length in *P. vulgaris.* (A-B) Primary root length in uninoculated **(A)** and *Ri*-inoculated **(B)** transgenic roots of control and *PvTOR*-RNAi at 12 days post inoculation. The statistical significance of differences between control and *PvTOR*-RNAi roots was determined using an unpaired two-tailed Student's *t*-test (*, $P < 0.05$). Error bars refer to the SD of the mean of three biological replicates (n > 30). *Ri*, *R. irregularis.*

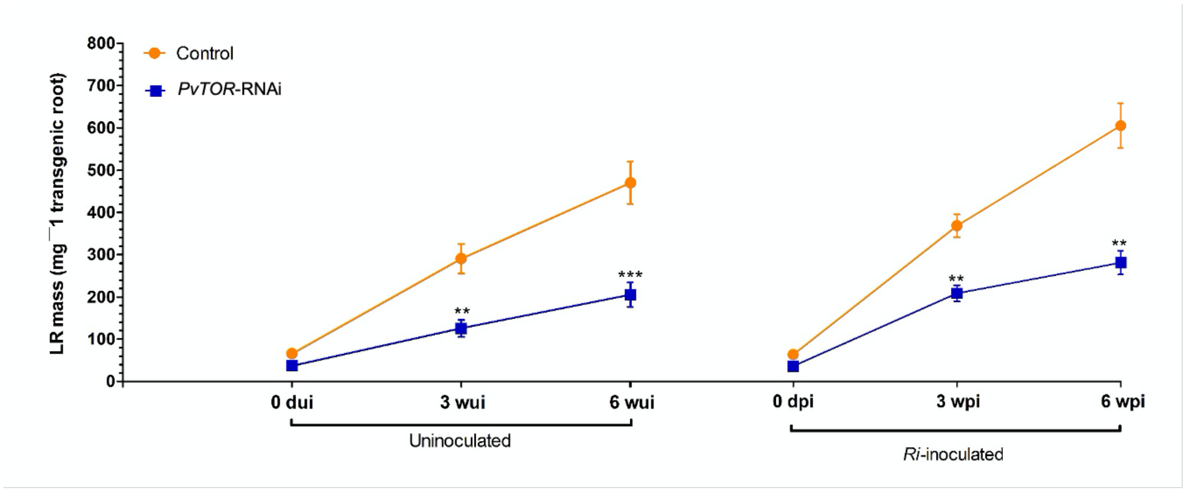

Figure S2. Quantitative analysis of lateral root mass in uninoculated and *Ri*-inoculated transgenic roots of control and *PvTOR*-RNAi at different time points. The statistical significance of differences between control and *PvTOR*-RNAi roots was determined using an unpaired two-tailed Student's *t*-test (**, $P < 0.01$; ***, $P < 0.001$). Error bars refer to the SE of the mean of three biological replicates (n > 30). LR, lateral root; *Ri*, *R. irregularis*; dui, day(s) uninoculated; dpi, day(s) post inoculation; wpi, week(s) post inoculation.

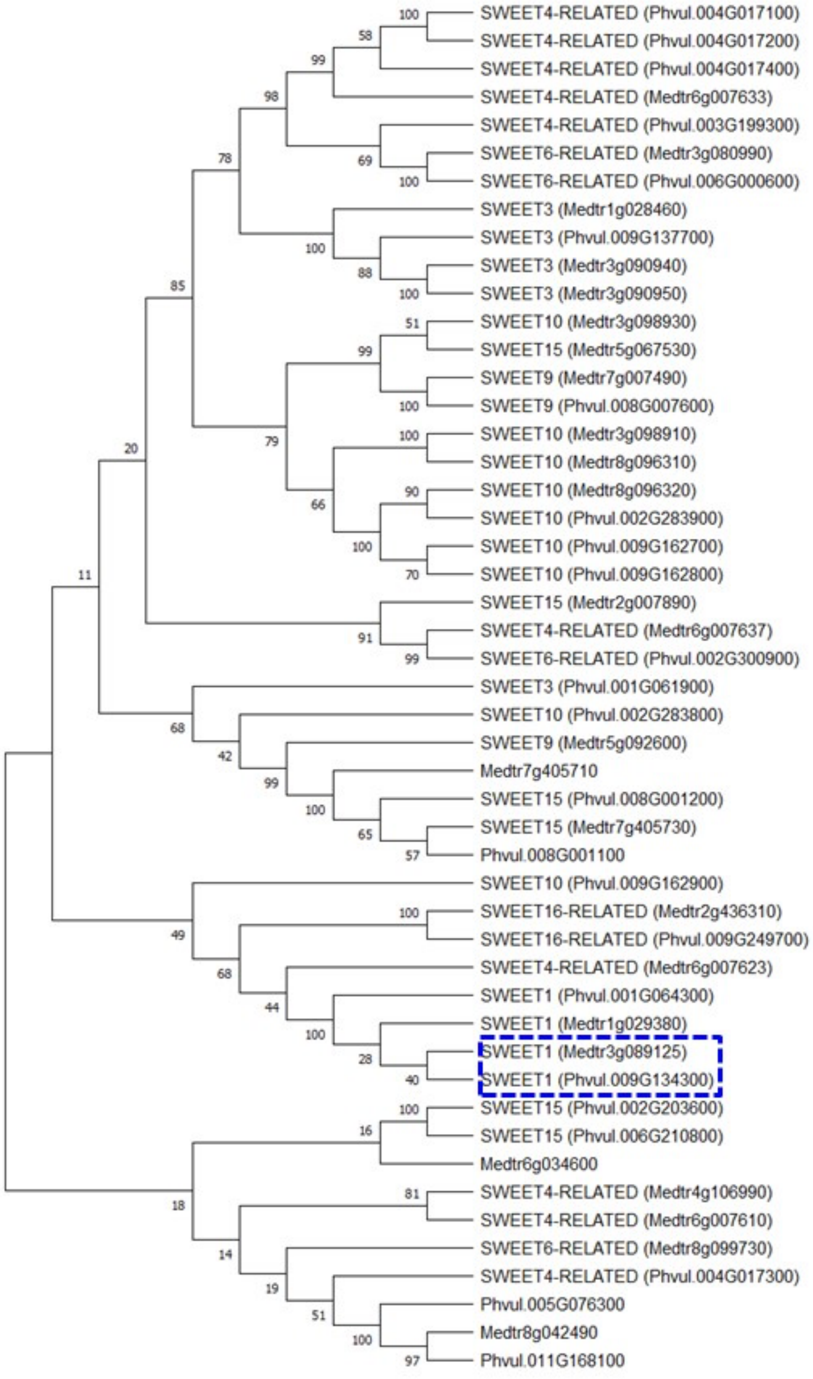

Figure S3. Phylogenetic analysis of *Phaseolus vulgaris* and *Medicago truncatula SWEET* genes. Protein sequences of SWEET homologs were identified in the Phytozome database. The phylogenetic tree was constructed using MEGA-X-10.1.1 software with the neighbor-joining (NJ) tree method with 1000 bootstrap values.

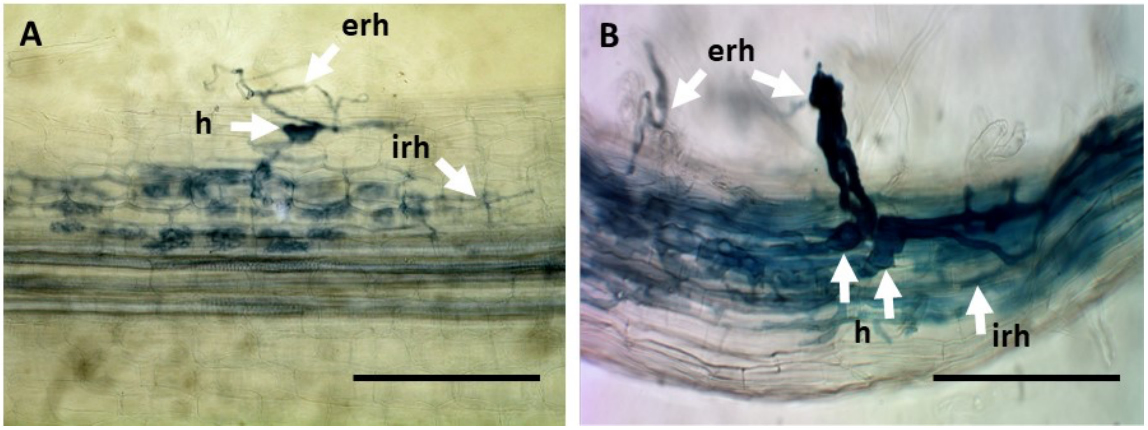

Figure S4. Micrograph of *R. irregularis*-inoculated transgenic roots showing hyphopodia at 3wpi. (A) Control and **(B)** *PvTOR*-RNAi. wpi, week(s) post inoculation; erh, extraradical hyphae; irh, intraradical hyphae; h, hyphopodia. Bars = 1 mm.

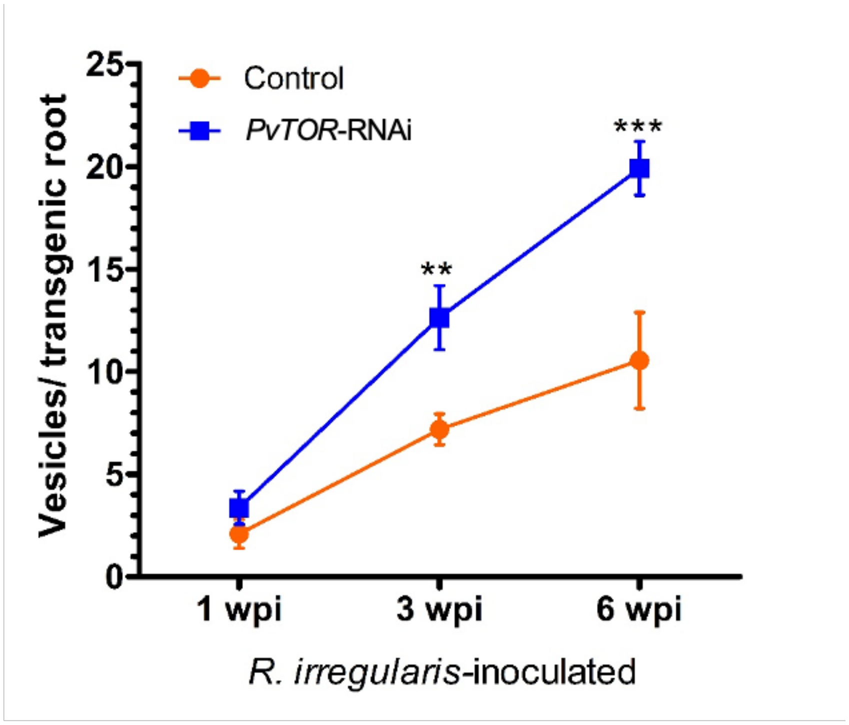

Figure S5. Quantification of vesicles in mycorrhized *P. vulgaris* control and *PvTOR*-RNAi roots at 1, 3 and 6 wpi. The statistical significance of differences between control and *PvTOR*-RNAi roots was determined using an unpaired two-tailed Student's *t*-test (**, $P < 0.01$; ***, $P < 0.001$). Error bars refer to the SD of the mean of three biological replicates (n > 30). wpi, week(s) post inoculation.

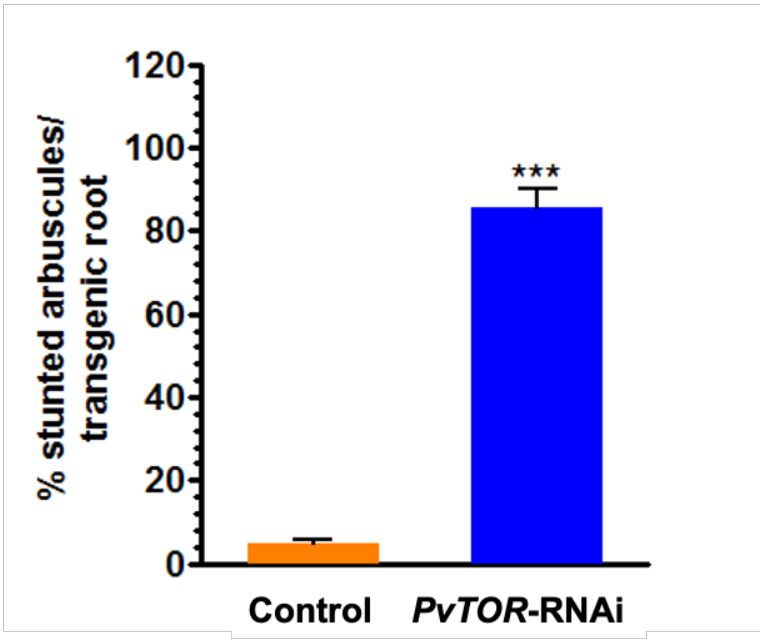

Figure S6. Quantification of stunted arbuscules per transgenic root of control and *PvTOR*-RNAi at 6 wpi with *R. irregularis*. The statistical significance of differences between control and *PvTOR*-RNAi roots was determined using an unpaired two-tailed Student's *t*-test (***, $P < 0.001$). Error bars refer to the SE of the mean of three biological replicates (n > 30). wpi, weeks post inoculation.

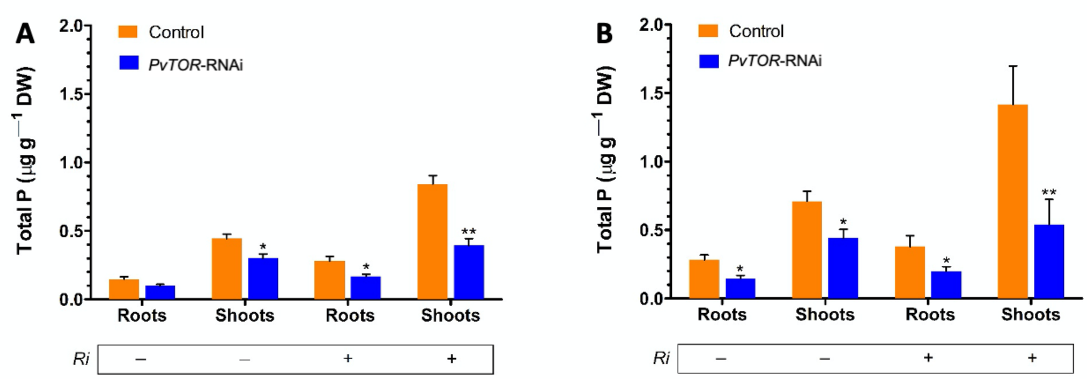

Figure S7. Total root and shoot phosphorus (P) concentration in mycorrhized composite plants of control and *PvTOR*-RNAi at **(A)** 3 wpi and **(B)** 6 wpi. The statistical significance of differences between control and *PvTOR*-RNAi samples was determined using an unpaired two-tailed Student's *t*-test (*, $P < 0.05$; **, $P < 0.01$). Error bars refer to the SD of the mean of three biological replicates (n > 12). wpi, week(s) post inoculation, Ri, *R. irregularis*.

Table S1: Primer sequences of *Phaseolus vulgaris* genes used to generate constructs and perform quantitative RT-PCR.

Gene		Oligonucleotide sequence (5' – 3')
EIF4a	F	GGTGTCCACACTGTTGTTGG
Phvul.010G136300	R	CGGCATTGTAGCAGAGAACA
IDE	F	GCAACCAACCTTTCATCAGC
Phvul.001G133200	R	AGAAATGCCTCAACCCTTTG
PvTOR-RNAi	F	CTTTCTGGTAATTCGCAAGGGAGCAGTA
Phvul.002G049900	R	AGCTAATTGAGGCATGCCCTTTCATCTG
PvTOR	F	GCTAGCCGTTTGTATTTTGGTGAGC
Phvul.002G049900	R	AGCAATTCCTGTCGATAGGCCTCA
UPB1	F	CAAAGAAGGGTGTTAGGAGGCCAAG
Phvul.007G089100	R	TGACACTCACTCTCGTTTGTAAGG
RGF6	F	GCTACAGGAAAATCAACAGCAGCAAG
Phvul.002G062100	R	CTGACCAGCTCCCAGCATTGACC
RGF9	F	AGGCCATGTCTTGTGCAAGTGCC
Phvul.002G267404	R	TTGTTGTGCAGCTTCATGTTGTGGC
GSS	F	GCATTGTTGCCGACGCCGTTTC
Phvul.006G094700	R	GACCGATGTAACCCCAATCGTATTTCCTC
APK2	F	GAAGGTTTTCAAGGTCATAAAGAGTGGTT
Phvul.002G089100	R	CCAATAGCCACTTTCATCCTCACTGA
SIR	F	TTGCTGACCAGTTTGGGATCGG
Phvul.011G021800	R	GATAATCCTTTCTCACGAGGGGAGC
APS1	F	TCTCCAAAACCTTCGACACC
Phvul.007G062900	R	AACCCACTGCAAGTCGATTC
APR3	F	GGCCTGTTTTACCTGGACAA
Phvul.003G079800	R	ATTTGCATGGGATTGGGATA
ORC5	F	CAACGGTGGACTGATGTCTG
Phvul.008G061800	R	CTCCTTGCAACCTTCAAAGC
ETG1	F	TCGCGGAATGATACAAGACA
Phvul.009G142700	R	CCGGGACACAGTAAAGCAGT
MCM7	F	GAAATGGCTAGGCACGTTGT
Phvul.001G212600	R	GAATGTGGAGCATTGGACCT
CDC6	F	AAAATCTCCCCACAGCTCCT
Phvul.006G064300	R	CTTGGCTCCACGAAAGTGAT
SUCS1	F	GGAAGCATGTGACCAACCTT
Phvul.009G223800	R	GTGAAAGCTGCATGAGACCA
SUCS2	F	CAAGTGAACCGTGCTCGTAA
Phvul.009G250800	R	AGGGCCACCATGACAAGTAG
CINV1	F	CTGGCTGTGATCCCAAAAAT
Phvul.007G091300	R	GCCACCAGATAACCTGCAAT

SWEET1	F	TGGAGTTCATGCCCTTCTTC
Phvul.009G134300	R	TCCATGAATTCCTCCTCTGC
SWEET6	F	CGCTTCTCTCCATTCTCACC
Phvul.006G000600	R	TGAAGGGATCAAAAGGGATG
SYMRK	F	GAAGATTTATGGTACTAGGT
Phvul.002G143400	R	TGTCAAGGCTACTCTGGA
CCAMK	F	GTGCATCACCAAGGAAGAAGTAG
Phvul.011G186900	R	TCACTGTTGGCATCCATTCTGTC
IPD3	F	GAAGAGGTGTGAAACCCTTGAGAA
Phvul.002G128600	R	AATCTCTTCAAGCAGACGCTCCTT
NSP2	F	ACTATTCGGCGGTGTTTGAC
Phvul.009G122700	R	TAGAAGCTTCGCTTGGCAAT
RAM1	F	GATATTGACCAAACTACCCTAACCCCACCA
Phvul.001G089900	R	CTAATTGGTCAGCAAAAAAGGATTCCCAC
RAM2	F	GCTTTTTCCACCTTCCCAACCGT
Phvul.007G233600	R	AACCCCACCAGCTTCAAAGG
VPY	F	ATCAAAGAACCCTCCTCCATGTTC
Phvul.001G113900	R	GAGCTTCTCTGATTTCATCTATGGAG
VAMP713	F	GGACTCACCGTTCTCTGCAT
Phvul.008G201600	R	ATTGATCCTGTCGGCGTTAG
STR	F	GGCCTGTACCTGATGGAGAA
Phvul.002G331200	R	CTTGGCTGCGTAGGCTAATC
STR2	F	TCGAGAAAGTGCACGACATC
Phvul.002G143300	R	TTCTCTCCCTTGGGGACTTT
PT-4	F	GCGGTGACTAACATGTTAGGG
Phvul.004G007300	R	CCTGTGCCCTAGTATTGTTGG
AM-1	F	GATGCAAGGTCAAAGGCCAC
Phvul.003G143400	R	AACACCAACCCTTCCCAACT
HA-1	F	TGGTAAACCAAAGGACCGCA
Phvul.010G050900	R	GCAGAGTGGAGGCCATGTAA
